# Associations of preterm and early-term birth with suspected developmental coordination disorder: a national retrospective cohort study in children aged 3–10 years

**DOI:** 10.1007/s12519-022-00648-9

**Published:** 2022-12-05

**Authors:** Ming-Xia Liu, Hai-Feng Li, Mei-Qin Wu, Shan-Shan Geng, Li Ke, Bi-Wen Lou, Wenchong Du, Jing Hua

**Affiliations:** 1 The Women’s and Children’s Department, Shanghai First Maternity and Infant Hospital, School of Medicine, Tongji University, Shanghai, China; 2 Shanghai Key Laboratory of Maternal Fetal Medicine, Shanghai First Maternity and Infant Hospital, School of Medicine, Tongji University, Shanghai, China; 3 Department of Rehabilitation, Children’s Hospital, Zhejiang University School of Medicine, National Clinical Research Center for Child Health, Hangzhou, China; 4 State Key Laboratory of Cognitive Neuroscience and Learning, Beijing Normal University, Beijing, China; 5 Leitontech Research Institution, Suzhou, China; 6 Department of Psychology, NTU Psychology, Nottingham Trent University, Nottingham, UK

**Keywords:** China, Early-term and preterm birth, Late motor impairment, Movement Assessment Battery-2 for Children

## Abstract

**Background:**

This study analyzed the motor development and suspected developmental coordination disorder of very and moderately preterm (< 34^+0^ gestational age), late preterm (34^+0^–36^+6^ gestational week), and early-term (37^+0^–38^+6^ gestational week) children compared to their full-term peers with a national population-based sample in China.

**Methods:**

A total of 1673 children (799 girls, 874 boys) aged 3–10 years old were individually assessed with the Movement Assessment Battery for Children-second edition (MABC-2). The association between gestational age and motor performance of children was analyzed using a multilevel regression model.

**Results:**

The global motor performance [*β* =  – 5.111, 95% confidence interval (CI) =  – 9.200 to – 1.022; *P* = 0.015] and balance (*β* =  – 5.182, 95% CI =  – 5.055 to – 1.158; *P* = 0.003) for very and moderately preterm children aged 3–6 years old were significantly lower than their full-term peers when adjusting for confounders. Late preterm and early-term children showed no difference. Moreover, very and moderately preterm children aged 3–6 years had a higher risk of suspected developmental coordination disorder (DCD) (≤ 5 percentile of MABC-2 score) when adjusting for potential confounders [odds ratio (OR) = 2.931, 95% CI = 1.067–8.054; *P* = 0.038]. Late preterm and early-term children showed no difference in motor performance from their full-term peers (each *P* > 0.05).

**Conclusions:**

Our findings have important implications for understanding motor impairment in children born at different gestational ages. Very and moderately preterm preschoolers have an increased risk of DCD, and long-term follow-up should be provided for early detection and intervention.

**Supplementary Information:**

The online version contains supplementary material available at 10.1007/s12519-022-00648-9.

## Introduction

Developmental coordination disorder (DCD) is a developmental disorder that is characterized by significant motor impairment, which commonly results in persistent difficulties when performing daily motor activities [1–3]. The prevalence of DCD in children is estimated to be 5%–6% worldwide, with a higher prevalence reported in China [4–6]. Preterm birth has been identified as a risk factor for DCD in children [7, 8]. Children born very preterm were found to have a higher prevalence of DCD [9–14]. Mild and moderate motor impairments were observed in nearly half of preterm children, including impairments in balance, manual dexterity and ball skills [15, 16]. It has been reported that very preterm infants (< 32 gestational weeks) [17] were at a higher risk of motor dysfunction, and most of these cases could be identified by age 3 [13]. In addition, the risk of gross and fine motor development was increased with the decrease in gestational age before 40 gestational weeks [18], and evidence showed that even late preterm (34–36 weeks) children experienced a neuromotor delay during the first year of life coupled with long-term adverse neurodevelopmental outcomes [18–20]. However, the literature showed inconsistent results, with some studies showing that late preterm infants (34–36 gestational weeks) were not different from full-born infants in their cognition, motor, behavior, and socioemotional development across childhood [21, 22]. More importantly, most of the previous studies used parent-filled subjective measurements to assess children’s motor performance, which may affect the accuracy of the results [23].

Moreover, the association of DCD with early-term birth should also be examined. According to the American Academy of Pediatrics, births occurring between 37 weeks 0 days and 38 weeks 6 days are defined as early term [24, 25]. Increasing evidence has reported that early-term births have adverse cognitive and academic performance compared to those born at 39 weeks or later [26–31]. The week of gestation in the full-term range from 37 to 40 weeks has also been associated with neuromotor and motor development in 9- to 15-week-old infants [32] and 12-month-old infants [30]. Recently, we first reported the association between early-term birth and suspected DCD using a questionnaire reported by parents [17]. However, little is known regarding long-term motor impairment in the early term beyond the preschool period (after 6 years old).

In this study, we used a national retrospective cohort study design and examined the association of preterm and early-term births with DCD based on an objective standardized test for DCD. We hypothesized that children born at very and moderately preterm, late preterm, and early term had an increased risk of DCD compared with full-term children. This study aimed to (1) describe the motor delays in children born at early-term (37^+0^–38^+6^ gestational weeks), late preterm (34^+0^–36^+6^ gestational weeks) and very and moderately preterm (< 34^+0^ gestational age) compared to full term; and (2) explore the effect of gestational age on motor impairment at preschool and school age.

## Methods

### Study participants

**Fig. 1:**
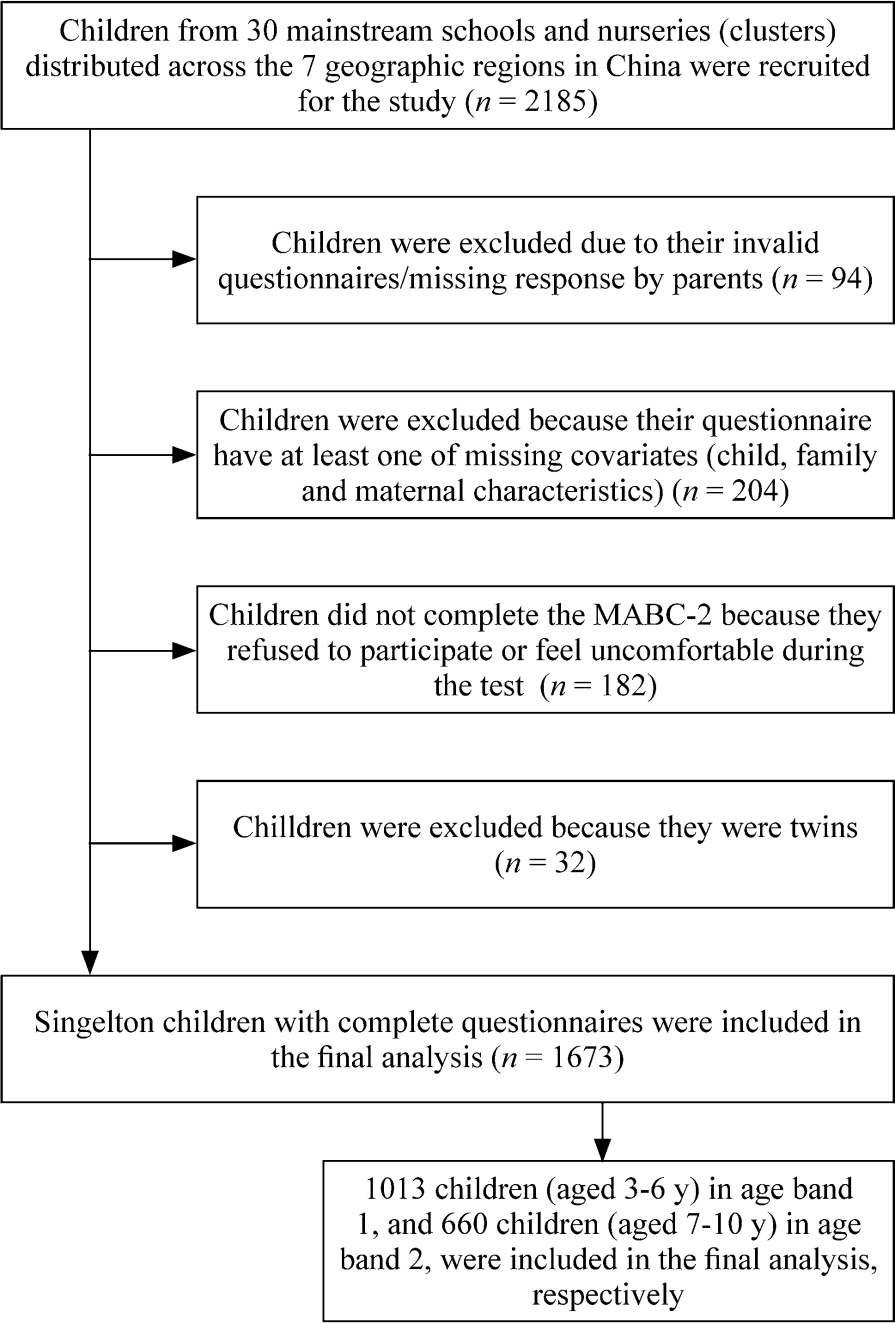
Flowchart of the study population. *MABC-2* Movement Assessment Battery-2 for Children

Children aged 3–10 years in urban China were recruited for the study. The 2010 National Census in China provided the basis for the stratification of the cluster sampling plan by geographic region, age, sex, and socioeconomic status. In addition, the sampling plan defined a group structure that identified the appropriate number of children in each group, which was defined according to the seven categories of geographic region [northeast (*n* = 179, 8.2%), north (*n* = 367, 16.8%), northwest (*n* = 141, 6.5%), southwest (*n* = 197, 9.0%), central (*n* = 265, 12.1%), east (*n* = 701, 32.1%), south (*n* = 335, 15.3%)], two categories of sex, ten categories of age, and four categories of parental educational levels. A total of 2185 children from 30 mainstream schools and nurseries (clusters) distributed across the seven geographic regions were recruited for the study. According to local regulations, blind, deaf children or those with severe intellectual disabilities or developmental disorders (e.g., autism) are required to attend special education schools. These schools were not included in our study; therefore, children’s intelligence, vision and hearing were assumed to be normal and were not measured in this study. The mechanism of initiating labor has been suggested to be different between twin and singleton gestations [33, 34]; therefore, participants who were twins or had missing variables needed for the analysis were excluded from the analysis (Fig. 1). There were 1673 participants included in the final analysis.

### Measures

The age band 1 (3–6 years) and age band 2 (7–10 years) of a standardized assessment for DCD, Movement Assessment Battery for Children-second edition (MABC-2) were used to test children’s motor impairment in the study. There are eight tasks for each age band of the MABC-2, including three subtests: the manual dexterity subtest contains three tasks (posting coins/placing pegs; threading lace; drawing); aiming and catching subtest contains two tasks (ball/beanbag catching and throwing); and balance subtest contains three tasks (one or two leg balance; walking lines; jumping or hopping). The MABC-2 has been widely used in the Chinese population, and previous research indicated that Chinese children were able to complete all test items of the MABC-2 given a sufficient understanding of the test instructions and procedures, and the tasks, test instructions, and procedures outlined in the MABC-2 are suitable for children in Chinese [6]. The MABC-2 has been approved to be suitable for use in the Chinese population with good to excellent interrater and test–retest reliability and good content and criteria-related validity [6, 35]. The intraclass correlation coefficient of interrater and test–retest reliability for each test item of the MABC-2 was good (each above 0.8). Confirmatory factor analysis showed that the goodness-of-fit indices of the adjusted model were good (each above 0.9), indicating a satisfactory fit of the data to the model. The total score on the MABC-2 and Peabody Developmental Motor Scales-2 was correlated well (*r* = 0.631), suggesting fair criteria-related validity. A standard total test score and standard scores of the three subtests (manual dexterity, aiming and catching, and balance) of the MABC-2 can be obtained based on the Chinese local norm. These scores were then grouped as suspected DCD (at or below the 5th percentile of the total test score), at risk of DCD (between the 6th and 16th percentiles of the total test score) and typical performance (above the 16th percentile of the total test score), according to the MABC-2 manual.

Gestational age was determined according to the parent's responses to the online questionnaire. Information including personal characteristics, family characteristics (including family socioeconomic information and family structure), and maternal health-related factors (including maternal age and delivery mode), which may affect child motor development according to the literature [7, 8, 31, 36], was gathered from the parent questionnaire (Table 1). Family structures were classified into three types: a single-parent family refers to a family with one single parent; a nuclear family refers to a family with both parents; and an extended family refers to a family with both parents and grandparents, which is a traditional family structure in China. Body mass index (BMI) was calculated as the weight in kilograms divided by height in meters squared. A child with BMI > 18 was indicated as being overweight.

**Table 1:** The mean scores of MABC-2 by children and family characteristics in all participants (*n* = 1673)

Characteristics	Total score	*P*	Manual dexterity	*P*	Aiming and catching	*P*	Balance	*P*
Maternal age (y)								
≤ 24	78.97 (11.016)	0.235^a^	29.23 (5.644)	0.863^a^	19.84 (4.678)	0.700^a^	29.74 (5.415)	0.106^a^
25–34	80.10 (10.769)		29.40 (5.242)		20.04 (4.773)		30.49 (5.228)	
≥ 35	80.95 (9.080)		29.55 (5.026)		20.31 (4.641)		30.85 (5.014)	
Delivery mode								
Vaginal birth	80.33 (10.609)	0.277^a^	29.55 (5.142)	0.254^a^	20.08 (4.619)	0.709^a^	30.55 (5.082)	0.377^a^
Cesarean section	79.76 (10.775)		29.26 (5.387)		19.99 (4.862)		30.32 (5.373)	
Children’s age (y)								
3–6 (age band 1)	80.44 (10.426)	0.049^b*^	29.41 (5.359)	0.825^b^	20.19 (4.551)	0.096^b^	30.56 (5.235)	0.195^b^
7–10 (age band 2)	79.37 (11.100)		29.36 (5.159)		19.79 (5.043)		30.22 (5.256)	
Sex								
Boys	78.59 (10.813)	< 0.001^b‡^	28.55 (5.309)	< 0.001^b‡^	20.57 (4.711)	< 0.001^b‡^	29.28 (5.342)	< 0.001^b‡^
Girls	81.56 (10.365)		30.30 (5.092)		19.44 (4.727)		31.66 (4.836)	
BMI								
> 18	76.36 (11.418)	< 0.001^b‡^	27.59 (5.627)	< 0.001^b‡^	20.14 (5.149)	0.775^b^	28.55 (5.275)	< 0.001^b‡^
≤ 18	80.41 (10.580)		29.59 (5.212)		20.02 (4.717)		30.61 (5.209)	
Mother’s higher education								
No	79.90 (9.955)	0.779^b^	29.02 (5.048)	0.093^b^	20.25 (4.740)	0.270^b^	30.38 (4.877)	0.829^b^
Yes	80.06 (10.949)		29.52 (5.349)		19.96 (4.755)		30.44 (5.363)	
Father’s higher education								
No	80.20 (10.100)	0.701^b^	29.26 (5.189)	0.559^b^	20.25 (4.639)	0.284^b^	30.34 (4.950)	0.695^b^
Yes	79.96 (10.884)		29.43 (5.305)		19.96 (4.785)		30.45 (5.330)	
Mother’s occupation								
Management & skilled	80.57 (10.887)	0.306^a^	29.49 (5.402)	0.869^a^	20.06 (4.976)	0.428^a^	30.78 (5.127)	0.231^a^
Others	79.72 (10.694)		29.34 (5.197)		19.96 (4.676)		30.30 (5.308)	
Unemployed	80.53 (10.115)		29.46 (5.503)		20.51 (4.583)		30.21 (5.067)	
Father’s occupation								
Management & skilled	80.73 (10.529)	0.113^a^	29.60 (5.218)	0.424^a^	20.10 (4.752)	0.438^a^	30.82 (5.122)	0.079^a^
Others	79.67 (10.808)		29.30 (5.306)		20.02 (4.757)		30.21 (5.310)^a^	
nemployed	77.81 (8.207)		28.50 (5.428)		18.56 (4.351)		30.75 (4.091)	
Family per-capita income of every mon (RMB)^c^								
≥ 23,821	80.20 (10.597)	0.989^b^	29.46 (5.247)	0.924^b^	20.03 (4.796)	0.536^b^	30.53 (5.120)	0.437^b^
< 23,821	80.19 (11.049)		29.50 (5.512)		20.25 (4.749)		30.24 (5.380)	
Family structure								
Single-parent families	81.50 (9.083)	0.840^a^	28.89 (5.624)	0.906^a^	20.83 (5.238)	0.712^a^	30.56 (3.240)	0.909^a^
Nuclear families	80.00 (10.771)		29.42 (5.266)		20.07 (4.805)		30.48 (5.095)	
Extended families	80.01 (10.667)		29.37 (5.287)		19.97 (4.685)		30.37 (5.434)	

Data are presented as mean (SD). *MABC-2* Movement Assessment Battery-2 for Children, *SD* standard deviation, *RMB* Ren Min Bi (Chinese currency). ^a^One-way ANOVA; ^b^two independent *t* test; ^c^the national average family per-capita income of the year before the survey time. ^*^P < 0.05,  ^†^*P *< 0.001

### Procedure

All assessors had proficient experience in conducting psychological assessments with children in a similar age range, and all assessors were trained with a two-day training program and were qualified to individually administer the MABC-2 test. More information regarding the quality control of the data collection can be found in our previous publication [8, 37, 38]. All children were assessed individually in their nurseries or schools. The assessment of each child lasted approximately 30–40 minutes. The height and weight of each child were also measured by each assessor. The study was approved by the Institutional Review Board (IRB), School of Brain and Cognitive Sciences, Beijing Normal University. All information acquired was kept confidential and was only accessible by the researchers. Consent forms and instructions for distribution to children were provided to the participating nurseries and schools. Consent was obtained from both participating nurseries and schools, as well as the children’s parents.

### Statistical analysis

One-way ANOVA was used to compare the mean scores of the MABC-2 based on the child and family characteristics. Chi-square analyses were used to compare the children and family characteristics among children with and without motor impairment. If the mean scores of the MABC-2 based on the child and family characteristics were significantly different, these variables were then considered potential confounders in the regression model.

The mixed model was used to investigate the associations of gestational age with MABC-2 scores when the clusters (nurseries or schools) and other potential confounders were adjusted for the potential confounders. Adjusted odds ratios were estimated to determine the strength of association for gestational age associated with poor motor performance (0 = typical performance with MABC-2 > 16 percentile; 1 = at-risk of motor impairment with MABC-2 of 6–16 percentile, 2 = significant motor impairment with MABC-2 < 6 percentile) using a multilevel logistic regression model. Analyses were carried out using MIXED, NLMIXED and GLIMMIX procedures of SAS 9.2 software, and *P* < 0.05 was denoted as statistically significant.

## Results

**Fig. 2:**
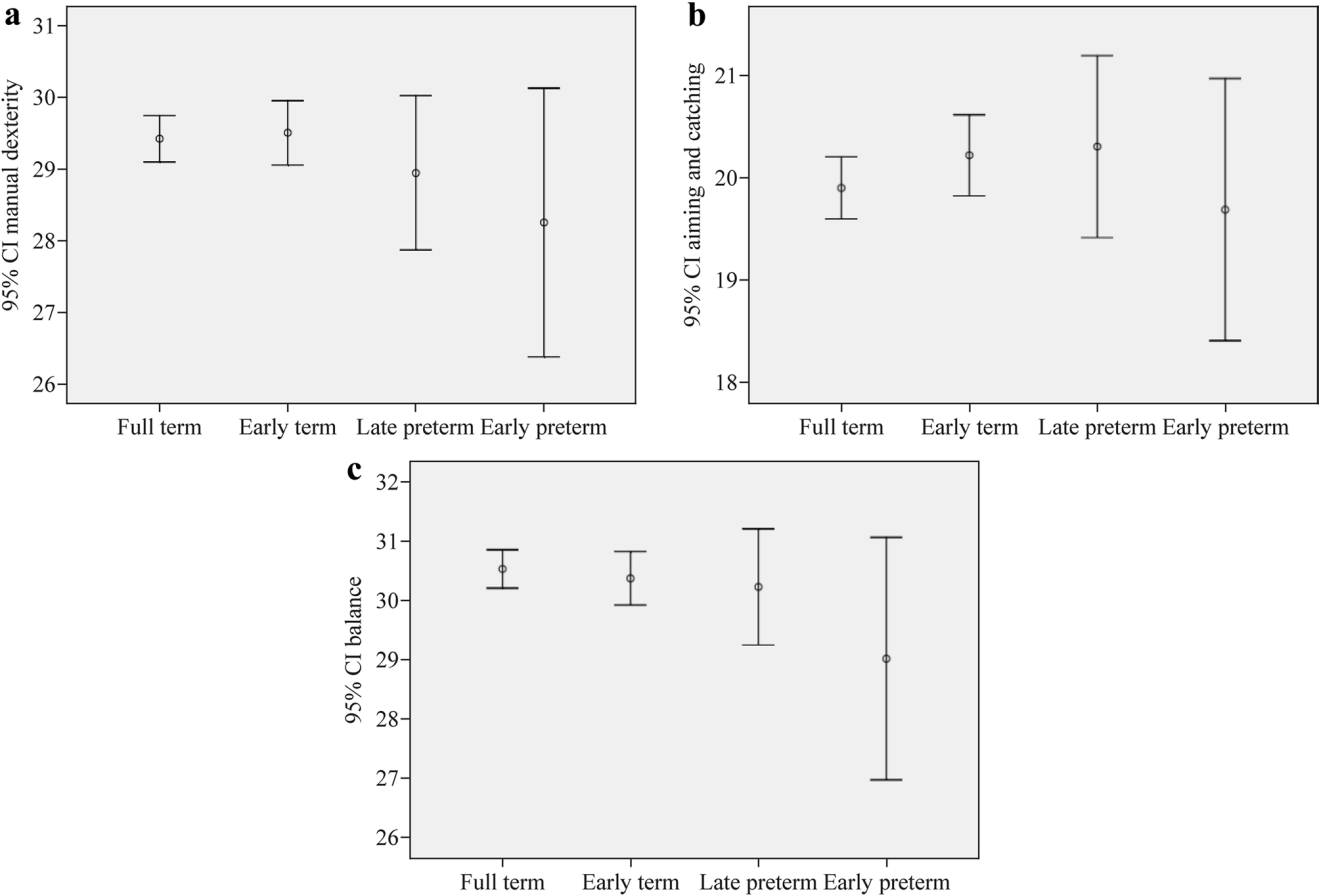
The mean scores of Movement Assessment Battery-2 for Children and 95% confidence intervals (CIs) in subjects (*n* = 1673). **a** Manual dexterity with 95% CI; **b** aiming and catching with 95% CI; **c** balance with 95% CI

Of the 1673 children (799 girls, 874 boys) included in the final analysis, 975 (58.3%) were full-term births, 542 (32.4%) were early-term births, 117 (7.0%) were late preterm births, and 39 (2.3%) were very and moderately preterm births. In children aged 3–6 years old (age band 1), the total scores of MABC-2 and subscores of balance in very and moderately preterm children were significantly lower than their full-term counterparts (*P* < 0.05). The mean total scores of MABC-2 with a 95% confidence interval (CI) by gestational age in all participants (*n* = 1673) and children of age band 1 (*n* = 1013) and age band 2 (*n* = 660), respectively, are shown in Fig. 2.

In all participants, the mean scores for manual dexterity, balance and total scores of the MABC-2 were higher in girls than in boys (each *P* < 0.001, Table 1). However, the mean score for aiming and catching was higher in boys than in girls (*P* < 0.001, Table 1). Children with a BMI of more than 18 scored lower in manual dexterity, balance and total scores of MABC-2 than children with a BMI of or less than 18 (*P* < 0.001, Table 1). Additionally, the rates of suspected DCD (≤ 5th of MABC-2) and at risk of DCD (6-16th of MABC-2) were distributed differently among different sex and BMI groups (Table 2). More boys than girls scored in the ≤ 5th and 6–16th percentiles on the MABC-2, and children with a BMI greater than 18 shared higher rates of suspected DCD and at risk of DCD. Similar distributions are shown in Supplementary Tables 1–4 when the participants were stratified by age band 1 (3–6 years old) and age band 2 (7–9 years old). The above differing variables were considered potential confounders and adjusted for in the following multilevel models.

**Table 2:** The rates of motor impairment by children and family’s characteristics in all participants (*n* = 1673)

Characteristics		MABC-2			
	Total	≤ 5 percentile (suspected DCD)	6–16 percentile (at risk of DCD)	> 16 percentile (typical performance)	*P*
Maternal age (y)					
≤ 24	205 (12.3)	13 (6.3)	28 (13.7)	164 (80.0)	0.304^a^
25–34	1358 (81.2)	79 (5.8)	154 (11.3)	1125 (82.8)	
≥ 35	110 (6.6)	2 (1.8)	16 (14.5)	92 (83.6)	
Delivery mode					
Vaginal birth	766 (45.8)	39 (5.1)	90 (11.7)	637 (83.2)	0.680^a^
Cesarean section	907 (54.2)	55 (6.1)	108 (11.9)	744 (82.0)	
Children’s age (y)					
3–6 (age band 1)	1013 (60.6)	53 (5.2)	108 (10.7)	852 (84.1)	0.109^a^
7–10 (age band 2)	660 (39.5)	41 (6.2)	90 (13.6)	529 (80.2)	
Sex					
Boys	874 (52.2)	62 (7.1)	126 (14.4)	686 (78.5)	< 0.001^a‡^
Girls	799 (47.8)	32 (4.0)	72 (9.0)	695 (87.0)	
BMI					
> 18	163 (9.7)	16 (9.8)	30 (18.4)	117 (71.8)	0.001^a†^
≤ 18	1500 (89.7)	78 (5.2)	168 (11.2)	1254 (83.6)	
Mother’s higher education					
No	429 (25.6)	19 (4.4)	50 (11.7)	360 (83.9)	0.447^a^
Yes	1244 (74.4)	75 (6.0)	148 (11.9)	1021 (82.1)	
Father’s higher education					
No	397 (23.7)	16 (4.0)	43 (10.8)	338 (85.1)	0.203^a^
Yes	1276 (76.3)	78 (6.1)	155 (12.1)	1043 (81.7)	
Mother’s occupation					
Management and skill	454 (27.1)	27 (5.9)	43 (9.5)	384 (84.6)	0.287^a^
Others	1077 (64.4)	62 (5.8)	139 (12.9)	876 (81.3)	
Unemployed	142 (8.5)	5 (3.5)	16 (11.3)	121 (85.2)	
Father’s occupation					
Management and skill	573 (34.3)	31 (5.4)	58 (10.1)	484 (84.5)	0.410^b^
Others	1084 (64.8)	63 (5.8)	137 (12.6)	884 (81.5)	
Unemployed	16 (1)	0 (0.0)	3 (18.8)	13 (81.3)	
Family per-capita income of every month (RMB)^c^					
≥ 23,821	1452 (86.8)	81 (5.6)	168 (11.6)	1203 (82.9)	0.670^a^
< 23,821	221 (13.2)	13 (5.9)	30 (13.6)	178 (80.5)	
Family structure					
Single families	18 (1.1)	0 (0.0)	3 (16.7)	15 (83.3)	0.694^b^
Nuclear families	859 (51.3)	47 (5.5)	108 (12.6)	704 (82.0)	
Extended families	796 (47.6)	47 (5.9)	87 (10.9)	662 (83.2)	

Data are presented as *n* (%). *MABC-2* Movement Assessment Battery-2 for Children, *DCD* developmental coordination disorder, *RMB* Ren Min Bi (Chinese currency). ^a^Pearson Chi-square test; ^b^Fisher exact test; ^c^the national average family per-capita income of the year before the survey time. ^†^*P* < 0.01, ^‡^*P* < 0.001

### Associations of gestational age with MABC-2 scores

In all participating children, we did not find delayed motor performance in early-term, late preterm or very and moderately preterm children (each *P* > 0.05, Table 3). In children aged 3–6 years old (age band 1), the total scores of MABC-2 (global motor performance) for very and moderately preterm children were significantly lower than those for full-term children (born at 39–41 gestational weeks) when not adjusting for [*β* =  – 5.476, 95% confidence interval (CI) =  – 9.671 to – 1.280; *P* = 0.012] or adjusting for potential confounders (*β* =  – 5.111, 95% CI =  – 9.200 to – 1.022; *P* = 0.015) using the mixed regression model. The subscores of balance for very and moderately preterm children were significantly lower than those for full-term children when not adjusting for (*β* =  – 3.437, 95% CI =  – 5.520 to – 1.354; *P* = 0.002) or adjusting for potential confounders (*β* =  – 5.182, 95% CI =  – 5.055 to – 1.158; *P* = 0.003). However, there was no statistically significant difference among the gestational ages in all participating children and in children aged 7–10 (each *P* > 0.05), which are shown in Table 3.

**Table 3:** Associations of gestational age with scores of MABC-2 (*n* = 1673)

Gestational age (wk)	Total score		Manual dexterity		Aiming and catching		Balance	
	*β*^a^ (95% CI)	*β*^b^ (95% CI)	*β*^a^ (95% CI)	*β*^b^ (95% CI)	*β*^a^ (95% CI)	*β*^b^ (95% CI)	*β*^a^ (95% CI)	*β*^b^ (95% CI)
Total (*n* = 1673)								
Full term (39–41)	Reference	Reference	Reference	Reference	Reference	Reference	Reference	Reference
Early term (37–38)	– 0.159 ( – 1.302, 0.984)	– 0.115 ( – 1.017, 1.247)	0.002 ( – 0.560, 0.563)	0.139 ( – 0.415, 0.692)	0.190 ( – 0.317, 0.698)	0.115 ( – 0.390, 0.620)	– 0.288 ( – 0.844, 0.268)	– 0.108 ( – 0.648, 0.433)
Late preterm (34–36)	– 0.440 ( – 2.505, 1.624)	– 0.009 ( – 2.035, 2.502)	– 0.409 ( – 1.422, 0.603)	– 0.212 ( – 1.210, 0.786)	0.412 ( – 0.505, 1.329)	0.290 ( – 0.622, 1.202)	– 0.211 ( – 1.214, 0.791)	0.043 ( – 0.931, 1.016)
Very and moderately preterm (< 34)	– 2.187 ( – 5.637, 1.262)	– 2.147 ( – 5.551, 1.258)	– 0.995 ( – 2.687, 0.697)	– 0.969 ( – 2.634, 0.696)	– 0.172 ( – 1.704, 1.360)	– 1.195 ( – 1.720, 1.327)	– 1.375 ( – 3.050, 0.301)	– 1.350 ( – 2.973, 0.274)
Age band 1 (*n* = 1013)								
Full term (39–41)	Reference	Reference	Reference	Reference	Reference	Reference	Reference	Reference
Early term (37–38)	– 1.082 ( – 2.495, 0.332)	– 0.715 ( – 2.096, 0.666)	– 0.246 ( – 0.971, 0.480)	– 0.056 ( – 0.768, 0.657)	– 0.094 ( – 0.712, 0.525)	– 0.133 ( – 0.753, 0.487)	– 0.570 ( – 1.273, 0.133)	– 0.349 ( – 1.029, 0.332)
Late preterm (34–36)	– 0.875 ( – 3.538, 1.787)	– 0.077 ( – 2.678, 2.525)	– 0.771 ( – 2.138, 0.595)	– 0.377 ( – 1.721, 0.966)	0.996 ( – 0.170, 2.162)	0.909 ( – 0.260, 2.079)	– 0.694 ( – 2.016, 0.629)	– 0.220 ( – 1.500, 1.060)
Very and moderately preterm (< 34)	– 5.476 ( – 9.671, – 1.280)^*^	– 5.111 ( – 9.200, – 1.022)^*^	– 1.838 ( – 3.991, 0.315)	– 1.606 ( – 3.180, 0.505)	– 0.733 ( – 2.571, 1.104)	– 0.809 ( – 2.647, 1.028)	– 3.437 ( – 5.520, – 1.354)^‡^	– 5.182 ( – 5.055, – 1.158)^‡^
Age band 2 (*n* = 660)								
Full-term (39–41)	Reference	Reference	Reference	Reference	Reference	Reference	Reference	Reference
Early term (37–38)	1.325 ( – 0.675, 3.325)	1.441 ( – 0.549, 3.431)	0.409 ( – 0.518, 1.336)	0.487 ( – 0.429, 1.403)	0.636 ( – 0.267, 1.542)	0.538 ( – 0.350, 1.426)	0.119 ( – 0.825, 1.064)	0.248 ( – 0.668, 1.163)
Late preterm (34–36)	0.296 ( – 3.111, 3.703)	0.213 ( – 3.177, 3.603)	0.091 ( – 1.483, 1.665)	0.102 ( – 1.453, 1.657)	– 0.267 ( – 1.808,1.273)	– 0.305 ( – 1.813, 1.204)	0.392 ( – 1.213, 1.997)	0.347 ( – 1.208, 1.903)
Very and moderately preterm (< 34)	3.631 ( – 2.596, 9.858)	3.104 ( – 3.100, 9.308)	0.569 ( – 2.307, 3.445)	0.192 ( – 2.654, 3.031)	0.856 ( – 1.959, 3.672)	1.311 ( – 1.452, 4.074)	2.197 ( – 0.737, 5.131)	1.594 (1.253, 4.440)^*^

*MABC-2* Movement Assessment Battery-2 for Children, *CI* confidence interval, *BMI* body mass index. ^a^No adjusted for other variables; ^b^adjusted for children’s age, sex, and BMI. ^*^P < 0.05, ^‡^*P* < 0.001

### Associations of gestational age with motor impairment

In all participants, the risk of suspected DCD increased in very and moderately preterm children when not adjusting for [odds ratio (OR) = 2.943, 95% CI = 1.087–7.974; *P* = 0.035] or adjusting for potential confounders (OR = 2.931, 95% CI = 1.067–8.054; *P* = 0.038), as shown in Table 4. In the participants aged 3–6 (age band 1), very and moderately preterm birth was associated with significant motor impairment when adjusting for potential confounders (OR = 3.673, 95% CI = 1.072–12.585; *P* = 0.040). However, other results without statistically significant differences (each *P* > 0.05) are shown in Table 4.

**Table 4:** Associations of gestational age with motor impairment (*n* = 1673)

Characteristics	MABC-2		At risk of DCD vs. typical performance			Suspected DCD vs. typical performance	
	≤ 5 percentile (suspected DCD), *n* (%)	6–16 percentile (at risk of DCD), *n* (%)	> 16 percentile (typical performance), *n* (%)	aOR (95% CI)	cOR (95% CI)	aOR (95% CI)	cOR (95% CI)
Total (*n* = 1673)							
Full-term (39–41)	116 (11.9)	48 (4.9)	811 (83.2)	Reference	Reference	Reference	Reference
Early-term (37–38)	64 (11.8)	33 (6.1)	445 (82.1)	1.239 (0.869, 1.765)	1.187 (0. 831, 1.696)	0.878 (0.517, 1.491)	0.820 (0.481, 1.398)
Late preterm (34–36)	11 (9.4)	9 (7.7)	97 (82.9)	1.250 (0.667, 2.343)	1.176 (0.625, 2.210)	1.492 (0.674, 3.343)	1.387 (0.618, 3.073)
Very and moderately preterm (< 34)	6 (15.4)	3 (7.7)	30 (76.9)	0.764 (0.209, 2.799)	0.764 (0.208, 2.810)	2.943 (1.087, 7.974)^*^	2.931 (1.067, 8.054)^*^
Age band 1 (*n* = 1013)							
Full term (39–41)	58 (10.0)	25 (4.3)	495 (85.6)	Reference	Reference	Reference	Reference
Early term (37–38)	40 (11.6)	21 (6.1)	283 (82.3)	1.238 (0.765, 2.003)	1.116 (0.693, 1.796)	1.469 (0.756, 2.851)	0.844 (0.426, 1.672)
Late preterm (34–36)	5 (7.6)	4 (6.1)	57 (86.4)	0.721 (0.249, 2.087)	1.176 (0.499, 2.765)	1.390 (0.413, 4.670)	1.365 (0.472, 3.949)
Very and moderately preterm (< 34)	5 (20.0)	3 (12.0)	17 (68.0)	2.286 (0.718, 7.286)	0.370 (0.038, 3.585)	3.494 (0.831, 14.634)	3.673 (1.072, 12.585)^*^
Age band 2 (*n* = 660)							
Full term (39–41)	58 (14.6)	23 (5.8)	316 (79.6)	Reference	Reference	Reference	Reference
Early term (37–38)	24 (12.2)	12 (6.1)	162 (81.8)	0.741 (0.312, 1.760)	0.763 (0.319, 1.822)	0.450 (0.140, 1.443)	0.461 (0.143, 1.485)
Late preterm (34–36)	6 (11.8)	5 (9.8)	40 (78.4)	1.223 (0.323, 4.634)	1.246 (0.326, 4.770)	1.220 (0.272, 5.478)	1.268 (0.280, 5.754)
Very and moderately preterm (< 34)	1 (7.1)	0 (0.0)	13 (92.9)	0.467 (0.016, 13.345)	0.405 (0.014, 11.714)	0.608 (0.021, 17.541)	0.546 (0.019, 15.969)

*MABC-2* Movement Assessment Battery-2 for Children, *DCD* developmental coordination disorder, *CI* confidence interval, *BMI* body mass index, *aOR* adjusted odds ratio (adjusted for children’s age, sex and BMI), *cOR* crude odds ratio. ^*^P < 0.05

## Discussion

To our knowledge, this is the first nationwide study on gestational age and suspected DCD using an objective standardized test (MABC-2) in both preschool and school-aged children. We observed significantly delayed motor performance in very and moderately preterm preschool children aged 3–6 years old, who were also more likely to be at risk of DCD when compared to a full-term birth. However, we did not find an association between late preterm and early-term born children with DCD, which is inconsistent with our previous study based on a parent-fill scale in preschool children [17].

Our study showed that very and moderately preterm preschoolers aged 3–6 years old were at a higher risk of DCD, which is similar to previous studies that showed poor motor performance was common in very preterm children with very low birth weight [39, 40]. Consistent with other reports, an increased risk of motor impairment was reported in children born very preterm at or before 32 weeks [10, 41–43]. Previous studies showed that the risk of DCD was 6 to 8 times higher in children born before 32 weeks (very preterm) than in children born at full term [9], while the risk of DCD was three to four times higher in children born before 37 weeks [44]. Our study confirmed the results with objective standardized motor assessment that the degree of prematurity is associated with the severity and prevalence of adverse neurodevelopmental outcomes [45]. It should also be noted that preterm children aged 7–10 years old did not show a difference from their full-term peers in their incidence rate of motor impairment. One potential reason could be that motor impairment in very and moderately preterm children is mild and could be mediated by environmental influences or natural maturation. Future research should be conducted to further examine the trajectories of motor development of preterm birth children.

The mechanism underlying a higher risk of motor impairment in very and moderately preterm infants can be explained from different aspects. Preterm infants are born during a particularly vulnerable phase of brain development and are therefore at a significantly higher risk of suboptimal brain development and adverse neurodevelopmental outcomes, including motor, neurosensory, cognitive, and behavioral deficits [40, 46]. Previous studies have consistently reported an association of brain microstructure with motor impairments in preterm populations [39, 47–49]. Impaired cerebellar development is an important determinant of adverse motor outcomes in very preterm infants [48], and studies have reported an association between fine motor skills and the volume and maturity of the cerebellum, brainstem and gray matter [50]. White matter injury, disrupted white matter maturation, injury of the supratentorial structures, including intraventricular hemorrhage and periventricular leukomalacia, and neonatal changes in the corpus callosum or cerebellar volume in preterm infants are associated with motor development disorders [40, 47, 49, 51–53]. Cortical folding mainly takes place in the third trimester of pregnancy, and a shortened gestation may therefore have a negative impact [54]. Additionally, brain development occurs in very specific time orders [27]. There are also distinct differences between the intrauterine and extrauterine environments due to the presence of maternal and placental hormones, which may also affect the brain development of preterm-born children [30]. Moreover, the brain structures of very preterm infants in early and term magnetic resonance imaging have been correlated with their concurrent motor, neurological and neurobehavioral functions [55]. Except for the affected brain development of preterm children, insufficient lung functioning and alterations in sleep patterns that have been associated with preterm birth may also affect the motor development of children [38, 56]. Furthermore, a range of parental factors related to preterm childcaring, such as increased parental concern about preterm children, may reduce children’s participation in physical activities, which can also affect the motor development of preterm children. The mechanisms underlying the higher risk of motor impairment in very and moderately preterm birth should be further examined in future research.

Our results did not find a difference in motor performance in late preterm or early-term children compared to their full-term peers. Cognitive, language and other developmental delays have been found in children born before 39 gestational weeks [57]. Late preterm infants have delays in early intellectual development, and late prematurity has been shown to induce a distinct neuronal pattern of structural change that can persist into school age [58]. Evidence suggests that children born at late preterm were also observed to have an increased risk of adverse developmental outcomes and academic performance compared to their full-term peers [59]. Late preterm infants had a higher risk of neurological impairments than full-term infants, which can be explained by their brain immaturity and an increased vulnerability to injury caused by a shortened gestation [60]. Even early-term births were also observed to be negatively affected in their brain development because the brain’s development of neural connections for specific cognitive areas is still undertaken at 37–38 weeks in gestation [61]. Our results may suggest that the motor impairment in late preterm and early-term children is mild and may only be revealed when the sample size is large.

There are limitations to our study. First, it should be noted that children with severe visual, hearing, intellectual impairments or other severe developmental disorders who were required to attend special education schools/nurseries were not recruited in the current study. It has been reported that there is a strong association between preterm birth and special education needs in children with severe impairments [62, 63]; further study should also include children with special education needs and examine the risk of children with severe impairment across the full range of gestation. Moreover, we should also consider the possibility that there are other conditions, such as undiagnosed attention problems or communication difficulties, that may affect MABC-2 performance. In the current study, we did not conduct a diagnostic assessment of DCD but only used the MABC-2 to assess children’s movement performance, and not all poor performance as measured by the MABC-2 would be clinically diagnosed as DCD. Therefore, we used suspected DCD in the current study. In addition, it should also be noted that because the prevalence of very and moderate preterm birth (gestational age earlier than 34 weeks) is relatively small in the population, there were only 39 children in the very and moderate preterm groups in our sample. However, despite a small sample with a wide confidence interval in the analysis, a significant result was found after adjusting for a wide range of confounders. Finally, based on a retrospective cohort, it is also difficult to conclude causal associations. Future investigation is needed to further examine the mechanisms underlying the association between a shortened gestation and motor impairment.

In conclusion, with a national sample and an objective standardized motor measurement for DCD, our study showed that children born before 34 gestational weeks had an increased risk of motor impairment when assessed at 3–6 years old. Our results emphasize the importance of long-term monitoring in children born very and moderately preterm, so early identification and intervention are needed to prevent adverse outcomes of motor impairment in these groups.

## Supplementary Information

Below is the link to the electronic supplementary material.

Supplementary file 1 (DOCX 58 KB)

## Data Availability

The datasets analyzed during the current study are available from the corresponding author upon reasonable request.
