# Supplementary material for: Associations of preterm and early-term birth with suspected developmental coordination disorder: a national retrospective cohort study in children aged 3–10 years
Source: World J Pediatr. 2022 Dec 5;19(3):261–72. doi: 10.1007/s12519-022-00648-9 (PMC9974676; doi:10.1007/s12519-022-00648-9)
Supplement: Supplementary file 1 — (DOCX 58 KB) [file 12519_2022_648_MOESM1_ESM.docx]

**Supplementary Table 1.** The mean scores of MABC-2 by children and family characteristics in children aged 3-6 years (*n* = 1013)

| Characteristics | Total score | *P* | Manual dexterity | *P* | Aiming and catching | *P* | Balance | *P* |
| --- | --- | --- | --- | --- | --- | --- | --- | --- |
| Maternal age^a^ (y) |  |  |  |  |  |  |  |  |
| ≤ 24 | 79.85 ± 10.237 | 0.844^a^ | 29.32 ± 5.799 | 0.982^a^ | 20.17 ± 4.395 | 0.961^a^ | 30.02 ± 4.841 | 0.569^a^ |
| 25-34 | 80.51 ± 10.578 |  | 29.43 ± 5.308 |  | 20.21 ± 4.555 |  | 30.61 ± 5.279 |  |
| ≥ 35 | 80.43 ± 8.939 |  | 29.39 ± 5.344 |  | 20.06 ± 4.691 |  | 30.65 ± 5.186 |  |
|  |  |  |  |  |  |  |  |  |
| Delivery mode^a^ |  |  |  |  |  |  |  |  |
| Vaginal birth | 80.93 ± 10.777 | 0.163^a^ | 29.68 ± 5.351 | 0.146^a^ | 20.20 ± 4.428 | 0.950^a^ | 30.80 ± 5.217 | 0.166^a^ |
| Caesarean section | 80.01 ± 10.077 |  | 29.19 ± 5.351 |  | 20.18 ± 4.653 |  | 30.35 ± 5.237 |  |
|  |  |  |  |  |  |  |  |  |
| Sex^b^ |  |  |  |  |  |  |  |  |
| Male | 78.69 ± 10.448 | < 0.001^b^ | 28.50 ± 5.517 | < 0.001^b^ | 20.47 ± 4.436 | 0.046^b^ | 29.43 ± 5.296 | < 0.001^b^ |
| Female | 82.31 ± 10.060 |  | 30.39 ± 4.999 |  | 19.90 ± 4.648 |  | 31.76 ± 4.884 |  |
|  |  |  |  |  |  |  |  |  |
| Over-weighted (BMI) ^b^ |  |  |  |  |  |  |  |  |
| No | 80.68 ± 10.376 | 0.003^b^ | 29.58 ± 5.271 | < 0.001^b^ | 20.18 ± 4.509 | 0.916^b^ | 30.65 ± 5.285 | 0.029^b^ |
| Yes | 76.68 ± 10.985 |  | 27.03 ± 6.219 |  | 20.25 ± 5.322 |  | 29.16 ± 4.498 |  |
|  |  |  |  |  |  |  |  |  |
| Mother’s higher education^b^ |  |  |  |  |  |  |  |  |
| No | 80.88 ± 9.290 | 0.501^b^ | 28.85 ± 5.234 | 0.125^b^ | 20.80 ± 4.438 | 0.050^b^ | 30.61 ± 4.682 | 0.893^b^ |
| Yes | 80.35 ± 10.640 |  | 29.53 ± 5.375 |  | 20.06 ± 4.561 |  | 30.55 ± 5.340 |  |
|  |  |  |  |  |  |  |  |  |
| Father’s higher education^b^ |  |  |  |  |  |  |  |  |
| No | 80.86 ± 9.394 | 0.507^b^ | 28.89 ± 5.495 | 0.139^b^ | 20.53 ± 4.217 | 0.254^b^ | 30.68 ± 4.651 | 0.737^b^ |
| Yes | 80.34 ± 10.638 |  | 29.53 ± 5.318 |  | 20.11 ± 4.617 |  | 30.53 ± 5.355 |  |
|  |  |  |  |  |  |  |  |  |
| Mother’s vocation^a^ |  |  |  |  |  |  |  |  |
| Management & skilled | 80.38 ± 10.164 | 0.674^a^ | 29.24 ± 5.486 | 0.761^a^ | 20.08 ± 4.556 | 0.414^a^ | 30.68 ± 5.025 | 0.730^a^ |
| Others | 80.35 ± 10.592 |  | 29.51 ± 5.312 |  | 20.17 ± 4.528 |  | 30.47 ± 5.370 |  |
| Unemployed | 81.46 ± 9.919 |  | 29.28 ± 5.247 |  | 20.84 ± 4.676 |  | 30.87 ± 4.812 |  |
|  |  |  |  |  |  |  |  |  |
| Father’s vocation^a^ |  |  |  |  |  |  |  |  |
| Management & skilled | 80.55 ± 10.585 | 0.757^a^ | 29.49 ± 5.440 | 0.560^a^ | 20.04 ± 4.629 | 0.595^a^ | 30.67 ± 5.300 | 0.833^a^ |
| Others | 80.42 ± 10.381 |  | 29.40 ± 5.298 |  | 20.29 ± 4.507 |  | 30.49 ± 5.214 |  |
| Unemployed | 78.18 ± 6.258 |  | 27.73 ± 5.901 |  | 19.45 ± 4.204 |  | 31.00 ± 3.924 |  |
|  |  |  |  |  |  |  |  |  |
| Family per-capita income of every mon (RMB) ^b^ |  |  |  |  |  |  |  |  |
| ≥ 23,821 | 80.53 ± 10.183 | 0.799^b^ | 29.52 ± 5.207 | 0.555^b^ | 20.15 ± 4.517 | 0.226^b^ | 30.59 ± 5.129 | 0.830^b^ |
| < 23,821 | 80.82 ± 11.805 |  | 29.11 ± 6.431 |  | 20.75 ± 4.863 |  | 30.47 ± 5.486 |  |
|  |  |  |  |  |  |  |  |  |
| Family structure^a^ |  |  |  |  |  |  |  |  |
| Single families | 85.86 ± 9.856 | 0.385^a^ | 28.57 ± 7.829 | 0.869^a^ | 22.43 ± 6.241 | 0.241^a^ | 31.71 ± 3.039 | 0.782^a^ |
| Nuclear families | 80.37 ± 10.759 |  | 29.48 ± 5.362 |  | 20.34 ± 4.628 |  | 30.48 ± 5.093 |  |
| Extended families | 80.43 ± 10.120 |  | 29.37 ± 5.322 |  | 20.03 ± 4.449 |  | 30.61 ± 5.370 |  |

Data are presented as mean ± standard deviation. *MABC-2* movement assessment battery-2 for children, *BMI* body mass index. ^a^One-way ANOVA; ^b^two independent *t* test

**Supplementary Table 2.** The mean scores of MABC-2 by children and family characteristics in children aged 7-9 years (*n* = 660)

| Characteristics | Total score | *P* | Manual dexterity | *P* | Aiming and catching | *P* | Balance | *P* |
| --- | --- | --- | --- | --- | --- | --- | --- | --- |
| Maternal age^a^ (y) |  |  |  |  |  |  |  |  |
| ≤ 24 | 78.18 ± 11.650 | 0.178^a^ | 29.15 ± 5.529 | 0.735^a^ | 19.55 ± 4.915 | 0.375^a^ | 29.49 ± 5.886 | 0.154^a^ |
| 25-34 | 79.46 ± 11.045 |  | 29.37 ± 5.140 |  | 19.77 ± 5.096 |  | 30.31 ± 5.144 |  |
| ≥ 35 | 82.46 ± 9.481 |  | 30.00 ± 4.000 |  | 21.04 ± 4.493 |  | 31.43 ± 4.509 |  |
|  |  |  |  |  |  |  |  |  |
| Delivery mode^a^ |  |  |  |  |  |  |  |  |
| Vaginal birth | 79.35 ± 10.274 | 0.969^a^ | 29.35 ± 4.782 | 0.980^a^ | 19.88 ± 4.917 | 0.673^a^ | 30.13 ± 4.832 | 0.715^a^ |
| Caesarean section | 79.39 ± 11.724 |  | 29.36 ± 5.445 |  | 19.71 ± 5.146 |  | 30.28 ± 5.573 |  |
|  |  |  |  |  |  |  |  |  |
| Gender^b^: |  |  |  |  |  |  |  |  |
| Male | 78.45 ± 11.356 | 0.025^b^ | 28.63 ± 4.986 | < 0.001^b^ | 20.74 ± 5.102 | < 0.001^b^ | 29.05 ± 5.412 | < 0.001^b^ |
| Female | 80.39 ± 10.737 |  | 30.15 ± 5.237 |  | 18.74 ± 4.770 |  | 31.51 ± 4.764 |  |
|  |  |  |  |  |  |  |  |  |
| Over-weighted (BMI) ^b^ |  |  |  |  |  |  |  |  |
| No | 79.95 ± 10.910 | 0.002^b^ | 29.62 ± 5.115 | 0.003^b^ | 19.75 ± 5.040 | 0.558^b^ | 30.56 ± 5.082 | < 0.001^b^ |
| Yes | 76.15 ± 11.732 |  | 27.94 ± 5.222 |  | 20.07 ± 5.064 |  | 28.17 ± 5.700 |  |
|  |  |  |  |  |  |  |  |  |
| Mother’s higher education^b^ |  |  |  |  |  |  |  |  |
| No | 79.22 ± 10.357 | 0.772^b^ | 29.14 ± 4.922 | 0.402^b^ | 19.87 ± 4.911 | 0.751^b^ | 30.22 ± 5.011 | 0.990^b^ |
| Yes | 79.47 ± 11.549 |  | 29.49 ± 5.303 |  | 19.74 ± 5.129 |  | 30.22 ± 5.409 |  |
|  |  |  |  |  |  |  |  |  |
| Father’s higher education^b^ |  |  |  |  |  |  |  |  |
| No | 79.60 ± 10.682 | 0.717^b^ | 29.58 ± 4.888 | 0.441^b^ | 20.00 ± 4.986 | 0.449^b^ | 30.03 ± 5.196 | 0.529^b^ |
| Yes | 79.27 ± 11.298 |  | 29.25 ± 5.282 |  | 19.69 ± 5.071 |  | 30.31 ± 5.287 |  |
|  |  |  |  |  |  |  |  |  |
| Mother’s vocation^a^ |  |  |  |  |  |  |  |  |
| Management & skilled | 80.91 ± 12.098 | 0.111^a^ | 29.93 ± 5.237 | 0.181^a^ | 20.02 ± 5.662 | 0.609^a^ | 30.96 ± 5.316 | 0.081^a^ |
| Others | 78.78 ± 10.788 |  | 29.09 ± 5.014 |  | 19.65 ± 4.879 |  | 30.06 ± 5.211 |  |
| Unemployed | 79.45 ± 10.306 |  | 29.67 ± 5.816 |  | 20.12 ± 4.477 |  | 29.45 ± 5.280 |  |
|  |  |  |  |  |  |  |  |  |
| Father’s vocation^a^ |  |  |  |  |  |  |  |  |
| Management & skilled | 81.07 ± 10.446 | 0.029^a^ | 29.80 ± 4.800 | 0.293^a^ | 20.21 ± 4.974 | 0.144^a^ | 31.08 ± 4.789 | 0.017^a^ |
| Others | 78.63 ± 11.312 |  | 29.14 ± 5.319 |  | 19.63 ± 5.068 |  | 29.82 ± 5.424 |  |
| Unemployed | 77.00 ± 12.390 |  | 30.20 ± 4.266 |  | 16.60 ± 4.450 |  | 30.20 ± 4.868 |  |
| Family per-capita income of every mon (RMB)^b^ |  |  |  |  |  |  |  |  |
| ≥ 23,821 | 79.63 ± 11.257 | 0.932^b^ | 29.37 ± 5.318 | 0.423^b^ | 19.84 ± 5.235 | 0.923^b^ | 30.41 ± 5.109 | 0.494^b^ |
| < 23,821 | 79.73 ± 10.489 |  | 29.78 ± 4.742 |  | 19.88 ± 4.649 |  | 30.06 ± 5.316 |  |
|  |  |  |  |  |  |  |  |  |
| Family structure^a^ |  |  |  |  |  |  |  |  |
| Single families | 78.73 ± 7.760 | 0.871^a^ | 29.09 ± 4.110 | 0.983^a^ | 19.82 ± 4.513 | 0.970^a^ | 29.82 ± 3.281 | 0.329^a^ |
| Nuclear families | 79.55 ± 10.782 |  | 29.35 ± 5.156 |  | 19.75 ± 4.992 |  | 30.47 ± 5.103 |  |
| Extended families | 79.12 ± 11.721 |  | 29.38 ± 5.221 |  | 19.85 ± 5.160 |  | 29.85 ± 5.544 |  |

Data are presented as mean ± standard deviation. *MABC-2* movement assessment battery-2 for children, *BMI* body mass index. ^a^One-way ANOVA; ^b^two independent *t* test

**Supplementary Table 3.** The rates of motor impairment by children and characteristics in total subjects in children aged 3-6 years

| Characteristics |  | MABC-2 | | MABC-2 > 16, typical performance | *P* |
| --- | --- | --- | --- | --- | --- |
|  |  | ≤ 5 percentile | 6-16 percentile |  |  |
| Maternal age (y) |  |  |  |  |  |
| ≤ 24 |  | 3 (3.1) | 16 (16.7) | 77 (80.2) | 0.147^a^ |
| 25-34 |  | 48 (5.7) | 81 (9.7) | 706 (84.6) |  |
| ≥ 35 |  | 2 (2.4) | 11 (13.4) | 69 (84.1) |  |
|  |  |  |  |  |  |
| Delivery mode |  |  |  |  |  |
| Vaginal birth |  | 26 (5.5) | 46 (9.7) | 403 (84.8) | 0.615^b^ |
| Caesarean section |  | 27 (5.0) | 62 (11.5) | 449 (83.5) |  |
|  |  |  |  |  |  |
| Gender |  |  |  |  |  |
| Male |  | 36 (6.8) | 70 (13.3) | 421 (79.9) | 0.001^b^ |
| Female |  | 17 (3.5) | 38 (7.8) | 431 (88.7) |  |
|  |  |  |  |  |  |
| Over-weighted (BMI) |  |  |  |  |  |
| No |  | 47 (5.0) | 97 (10.3) | 798 (84.7) | 0.041^a^ |
| Yes |  | 6 (9.5) | 11 (17.5) | 46 (73.0) |  |
|  |  |  |  |  |  |
| Mother’s higher education |  |  |  |  |  |
| No |  | 6 (3.4) | 16 (9.1) | 154 (87.5) | 0.338^b^ |
| Yes |  | 47 (5.6) | 92 (11.0) | 698 (83.4) |  |
|  |  |  |  |  |  |
| Father’s higher education |  |  |  |  |  |
| No |  | 5 (2.7) | 18 (9.6) | 165 (87.8) | 0.169^b^ |
| Yes |  | 48 (5.8) | 90 (10.9) | 687 (83.3) |  |
|  |  |  |  |  |  |
| Mother’s vocation |  |  |  |  |  |
| Management & skilled |  | 17 (5.8) | 23 (7.9) | 251 (86.3) | 0.172^a^ |
| Others |  | 35 (5.4) | 74 (11.5) | 537 (83.1) |  |
| Unemployed |  | 1 (1.3) | 11 (14.5) | 64 (84.2) |  |
|  |  |  |  |  |  |
| Father’s vocation |  |  |  |  |  |
| Management & skilled |  | 22 (6.0) | 34 (9.2) | 312 (84.8) | 0.729^a^ |
| Others |  | 31 (4.9) | 73 (11.5) | 530 (83.6) |  |
| Unemployed |  | 0 (0.0) | 1 (9.1) | 10 (90.9) |  |
| Family per-capita income of every mon (RMB) |  |  |  |  |  |
| ≥ 23,821 |  | 46 (5.0) | 97 (10.5) | 777 (84.5) | 0.462^a^ |
| < 23,821 |  | 7 (7.5) | 11 (11.8) | 75 (80.6) |  |
|  |  |  |  |  |  |
| Family structure |  |  |  |  |  |
| Single families |  | 0 (0.0) | 0 (0.0) | 7 (100.0) | 0.350^a^ |
| Nuclear families  Extended families |  | 25 (5.4)  28 (5.2) | 59 (12.7)  49 (9.0) | 380 (81.9)  465 (85.8) |  |

Data are presented as *n* (%). *MABC-2* movement assessment battery-2 for children, *BMI* body mass index. ^a^Fisher exact test; ^b^Pearson Chi-square test

**Supplementary Table 4.** The rates of motor impairment by children and characteristics in total subjects in children aged 7-9 years

| Characteristics |  | MABC-2 | | MABC-2 > 16, typical performance | *P* |
| --- | --- | --- | --- | --- | --- |
|  |  | ≤ 5 percentile | 6-16 percentile |  |  |
| Maternal age |  |  |  |  |  |
| ≤ 24 |  | 10 (9.2) | 12 (11.0) | 87 (79.8) | 0.383^a^ |
| 25-34 |  | 31 (5.9) | 73 (14.0) | 419 (80.1) |  |
| ≥ 35 |  | 0 (0.0) | 5 (17.9) | 23 (82.1) |  |
|  |  |  |  |  |  |
| Delivery mode |  |  |  |  |  |
| Vaginal birth |  | 13 (4.5) | 44 (15.1) | 234 (80.4) | 0.186^b^ |
| Caesarean section |  | 28 (7.6) | 46 (12.5) | 295 (79.9) |  |
|  |  |  |  |  |  |
| Gender |  |  |  |  |  |
| Male |  | 26 (7.5) | 56 (16.1) | 265 (76.4) | 0.037^b^ |
| Female |  | 15 (4.8) | 34 (10.9) | 264 (84.3) |  |
|  |  |  |  |  |  |
| Over-weighted (BMI) |  |  |  |  |  |
| No |  | 31 (5.6) | 71 (12.7) | 456 (81.7) | 0.041^b^ |
| Yes |  | 10 (10.0) | 19 (19.0) | 71 (71.0) |  |
|  |  |  |  |  |  |
| Mother’s higher education |  |  |  |  |  |
| No |  | 13 (5.1) | 34 (13.4) | 206 (81.4) | 0.651^b^ |
| Yes |  | 28 (6.9) | 56 (13.8) | 323 (79.4) |  |
|  |  |  |  |  |  |
| Father’s higher education |  |  |  |  |  |
| No |  | 11 (5.3) | 25 (12.0) | 173 (82.8) | 0.504^b^ |
| Yes |  | 30 (6.7) | 65 (14.4) | 356 (78.9) |  |
|  |  |  |  |  |  |
| Mother’s vocation |  |  |  |  |  |
| Management & skilled |  | 10 (6.1) | 20 (12.3) | 133 (81.6) | 0.555^a^ |
| Others |  | 27 (6.3) | 65 (15.1) | 339 (78.7) |  |
| Unemployed |  | 4 (6.1) | 5 (7.6) | 57 (86.4) |  |
|  |  |  |  |  |  |
| Father’s vocation |  |  |  |  |  |
| Management & skilled |  | 9 (4.4) | 24 (11.7) | 172 (83.9) | 0.200^a^ |
| Others |  | 32 (7.1) | 64 (14.2) | 354 (78.7) |  |
| Unemployed |  | 0 (0.0) | 2 (40.0) | 3 (60.0) |  |
| Family per-capita income of every mon (RMB) |  |  |  |  |  |
| ≥ 23,821 |  | 35 (6.6) | 71 (13.3) | 426 (80.1) | 0.678^b^ |
| < 23,821 |  | 6 (4.7) | 19 (14.8) | 103 (80.5) |  |
|  |  |  |  |  |  |
| Family structure |  |  |  |  |  |
| Single families |  | 0 (0.0) | 3 (27.3) | 8 (72.7) | 0.362^a^ |
| Nuclear families  Extended families |  | 22 (5.6)  19 (7.5) | 49 (12.4)  38 (15.0) | 324 (82.0)  197 (77.6) |  |

Data are presented as *n* (%).*MABC-2* movement assessment battery-2 for children, *BMI* body mass index. ^a^Fisher exact test; ^b^Pearson Chi-square test
